# Library of single-etch silicon nitride grating couplers for low-loss and fabrication-robust fiber-chip interconnection

**DOI:** 10.1038/s41598-023-44824-x

**Published:** 2023-10-14

**Authors:** Radovan Korček, David Medina Quiroz, Quentin Wilmart, Samson Edmond, Pavel Cheben, Laurent Vivien, Carlos Alonso-Ramos, Daniel Benedikovič

**Affiliations:** 1 Department of Multimedia and Information-Communication Technology, University of Zilina, 010 26 Žilina, Slovakia; 2 Centre de Nanosciences et de Nanotechnologies, CNRS, Université Paris-Saclay, 91120 Palaiseau, France; 3 Université Grenoble Alpes, CEA, LETI, 38000 Grenoble, France; 4 National Research Council Canada, Ottawa, ON K1A 0R6 Canada; 5 University Science Park, University of Zilina, 010 26 Žilina, Slovakia

**Keywords:** Micro-optics, Nanophotonics and plasmonics, Integrated optics

## Abstract

Silicon nitride (Si_3_N_4_) waveguides become an appealing choice to realize complex photonic integrated circuits for applications in telecom/datacom transceivers, sensing, and quantum information sciences. However, compared to high-index-contrast silicon-on-insulator platform, the index difference between the Si_3_N_4_ waveguide core and its claddings is more moderate, which adversely affects the development of vertical grating-coupled optical interfaces. Si_3_N_4_ grating couplers suffer from the reduced strength, therefore it is more challenging to radiate all the waveguide power out of the grating within a beam size that is comparable to the mode field diameter of standard optical fibers. In this work, we present, by design and experiments, a library of low-loss and fabrication-tolerant surface grating couplers, operating at 1.55 μm wavelength range and standard SMF-28 fiber. Our designs are fabricated on 400 nm Si_3_N_4_ platform using single-etch fabrication and foundry-compatible low-pressure chemical vapor deposition wafers. Experimentally, the peak coupling loss of − 4.4 dB and − 3.9 dB are measured for uniform couplers, while apodized grating couplers yield fiber-chip coupling loss of − 2.9 dB, without the use of bottom mirrors, additional overlays, and multi-layered grating arrangements. Beside the single-hero demonstrations, over 130 grating couplers were realized and tested, showing an excellent agreement with finite difference time domain designs and fabrication-robust performance. Demonstrated grating couplers are promising for Si_3_N_4_ photonic chip prototyping by using standard optical fibers, leveraging low-cost and foundry-compatible fabrication technologies, essential for stable and reproducible large-volume device development.

## Introduction

Integrated photonics is widely recognized as a pivotal technology that stimulates research and commercial drive in many applications. Over the recent years, integrated photonics, dominantly populated by the silicon-based (Si-based) material platforms, expands its frontiers towards fiber-optic communications, sensing, and quantum information sciences^1–3^. In this context, optical fibers have been a key development to form a backbone for communication systems in near-infrared (near-IR) wavebands, enabling large link capacities and high-speed data transmission. However, fiber dimensions are not compatible with sub-micrometer waveguide geometries used to guide and route the light in photonic integrated circuits. Small dimensions of photonic devices complicate optical chip interfacing with standard optical fibers due to the largely disparate mode sizes between optical fibers and on-chip waveguides.

Silicon-on-insulator (SOI) is established as mature platform to develop complex photonic circuits^4,5^, increasing the integration density, while reducing the production cost. However, SOI’s high-index contrast encompasses several drawbacks, including high sensitivity to fabrication imperfections, strong birefringence, and substantial scattering loss generated due to the nm-scaled sidewall waveguide roughness. Meanwhile, silicon nitride (Si_3_N_4_) photonics starts to play a leading role to supplement and/or even replace SOI platform. Si_3_N_4_ leverages superior passive functionalities, while keeping compatibility with complementary metal–oxide–semiconductor (CMOS) fabrication technology, which in turn, promises scalable, low-cost, and high-volume-produced optical building blocks^6–8^. The basic properties of Si_3_N_4_ films, such as thickness and refractive index, can be controlled by adjusting the deposition process of low-pressure chemical vapor deposition (LPCVD) or plasma-enhanced chemical vapor deposition (PECVD). This versatility also facilitates convenient control over the light confinement, dispersion, or polarization characteristics, with a Si_3_N_4_ transparency over wide wavelength range. To date, a rich library of Si_3_N_4_ photonic devices has been demonstrated. This includes low-loss waveguides^9^, filters^10^, splitters^11^, multiplexers^12^, ring resonators^13^, and grating and edge couplers^14–16^, among others. Compared to SOI waveguides, the index contrast between the Si_3_N_4_ light-guiding layer and the claddings is more moderate, typically ~ 0.5 (cladded with silica) and ~ 1.0 (cladded with air), hence it is challenging to obtain a low-loss interconnection between off-chip environment and SiN dies.

Grating couplers are an appealing structures to couple light in and out of photonic integrated circuits. The size of the waveguide mode is enlarged by forming a diffraction grating on a chip surface^17^, enabling flexible placing on a die and relaxed spatial alignment for fiber attachments. They are also compatible with planar manufacturing and automated on-wafer testing, which is one of key parts to advance photonic integration and packaging. However, Si_3_N_4_ grating couplers have higher coupling loss penalty than their SOI-based counterparts due to the moderately small index contrast, resulting in poor field overlap and low directionality^18^. Indeed, lower index contrast substantially reduces the strength of the individual scattering elements of the grating. This way, it is more difficult to radiate all the power from the waveguide over a fixed grating length and matching the out-radiated grating field to the mode size of conventional optical fiber. To enhance the fiber-chip coupling with Si_3_N_4_ couplers, several approaches have been reported^19–32^. This includes utilization of thicker waveguides^19^, adding bottom reflectors^20–25^, or depositing high-index material overlays^26–28^. Moreover, multi-layer topologies^29,30^, dual-etch geometries^31^, or adoption of hybrid Si_3_N_4_-on-SOI^32^ and Si-on-Si_3_N_4_^33^ platforms have been proposed and demonstrated recently. However, backside processing, wafer bonding, localized film deposition, or precise alignment between layers of different etches and materials call upon customized fabrication, which comes up with additional expenses. Consequently, this not only mandates complex processing and increased production cost, but in many cases requires dedicated services that are not widely available in today’s photonic foundries and their pilot-line fabrication runs^34^.

In this work, we develop a library of low-loss and fabrication-robust fiber-chip surface grating couplers realized on LPCVD-based Si_3_N_4_ photonic platform, with a low-cost single-etch manufacturing and Si-foundry-compatible processing. We show a comprehensive and general design process to implement Si_3_N_4_ grating couplers and full experimental characterization of various surface grating designs—more than 130 device flavors measured—with uniform and non-uniform geometries, demonstrating efficient and error-tolerant interconnection between optical fiber and Si_3_N_4_ chips.

### Design methodology and simulations

#### Uniform and apodized surface grating designs

**Figure 1:**
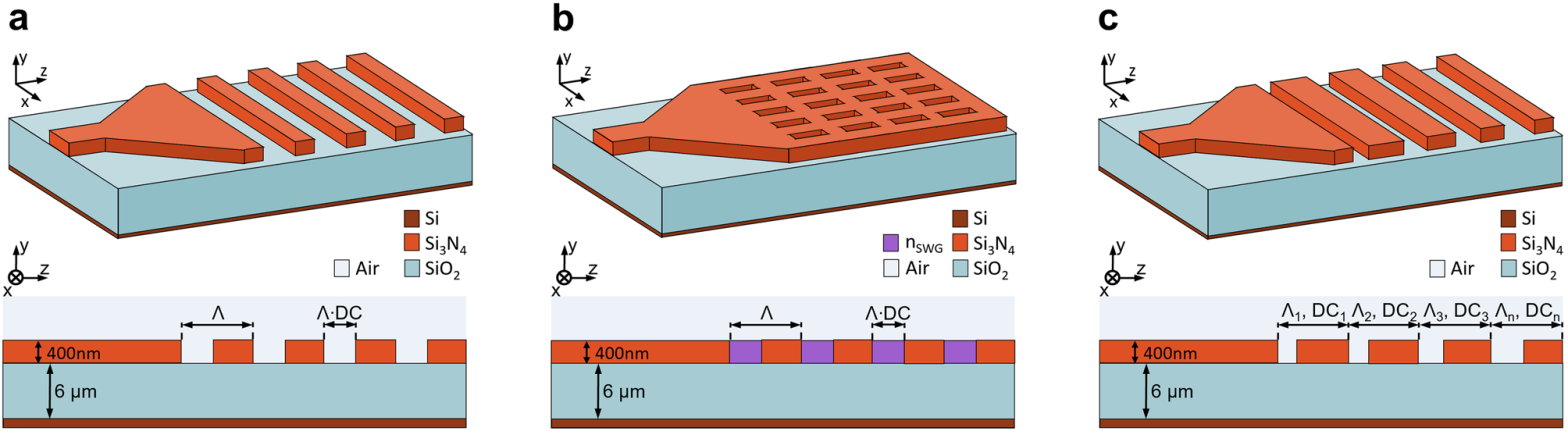
Silicon nitride surface grating couplers: uniform (**a**) without and (**b**) with sub-wavelength metamaterials, and (**c**) apodized designs.

Figure 1 shows three-dimensional (3-D) schematics of single-etch uniform and apodized grating couplers. The waveguide couplers are realized on a native Si_3_N_4_ photonic platform, comprising bottom silicon (Si) substrate, 6 μm thick buried oxide (BOX) layer, and 400 nm thick waveguide core, with an air as a superstrate medium. The refractive indexes of Si, SiN, silicon dioxide (SiO_2_), and air are^35^: *n*_Si_ = 3.476, *n*_Si3N4_ = 1.9902, *n*_SiO2_ = 1.444, and *n*_air_ = 1, respectively. The dispersion of individual materials is taken into the account. Grating couplers are designed to minimize the coupling loss between off-chip optical fiber (SMF-28) and a transverse electric (TE) waveguide mode at a central wavelength of 1.55 μm (*C*-band telecom range).

**Figure 2:**
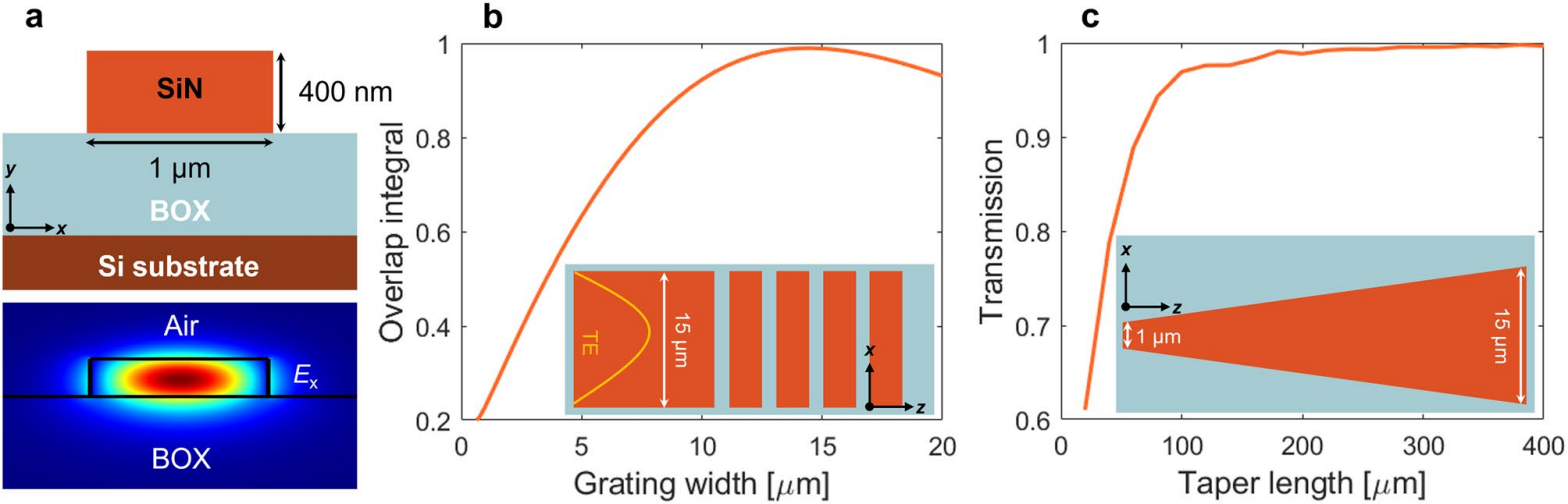
Interconnecting waveguide design. (**a**) Cross-section of a single-mode Si_3_N_4_ strip waveguide with a 2-D field profile for the fundamental TE mode. (**b**) Overlap integral as a function of the grating width between fiber mode profile and lateral *E*_x_ field component. (**c**) Transition efficiency as a function of the taper length, connecting 1 μm wide single-mode waveguide and 15 μm wide surface grating.

As schematically illustrated in Fig. 1a,c, waveguide couplers consist of surface grating fully etch down to the BOX and strip-like interconnecting waveguides. The single-mode Si_3_N_4_ waveguide, shown in Fig. 2a, is 400 nm thick and 1 μm wide. This waveguide is connected to a 15 μm wide surface grating using an adiabatic waveguide taper. The lateral grating width (see Fig. 2b) was optimized to have a near-unity overlap integral between the profile of the fiber mode and a dominant *E*_x_ component of the electric field. The optical fiber mode is modeled as a Gaussian function, with a 10.4 μm mode field diameter (MFD) defined at 1/e^2^ intensity and a wavelength of 1.55 μm. The waveguide-to-grating taper was calculated to be 400 μm long, providing a high transition efficiency of 99%, as shown in Fig. 2c.

The grating operation can be described through phase matching condition:

1 $$n_{c} \sin \Theta = n_{fb} + \frac{k\lambda }{\Lambda }$$

where *n*_c_ is the refractive index of cladding, *Θ* is the radiation angle, *n*_fb_ is the effective index of the TE-polarized Floquet–Bloch mode inside the grating, *k* is the diffraction order, *λ* is the operating wavelength, and *Λ* is the grating period.

**Figure 3:**
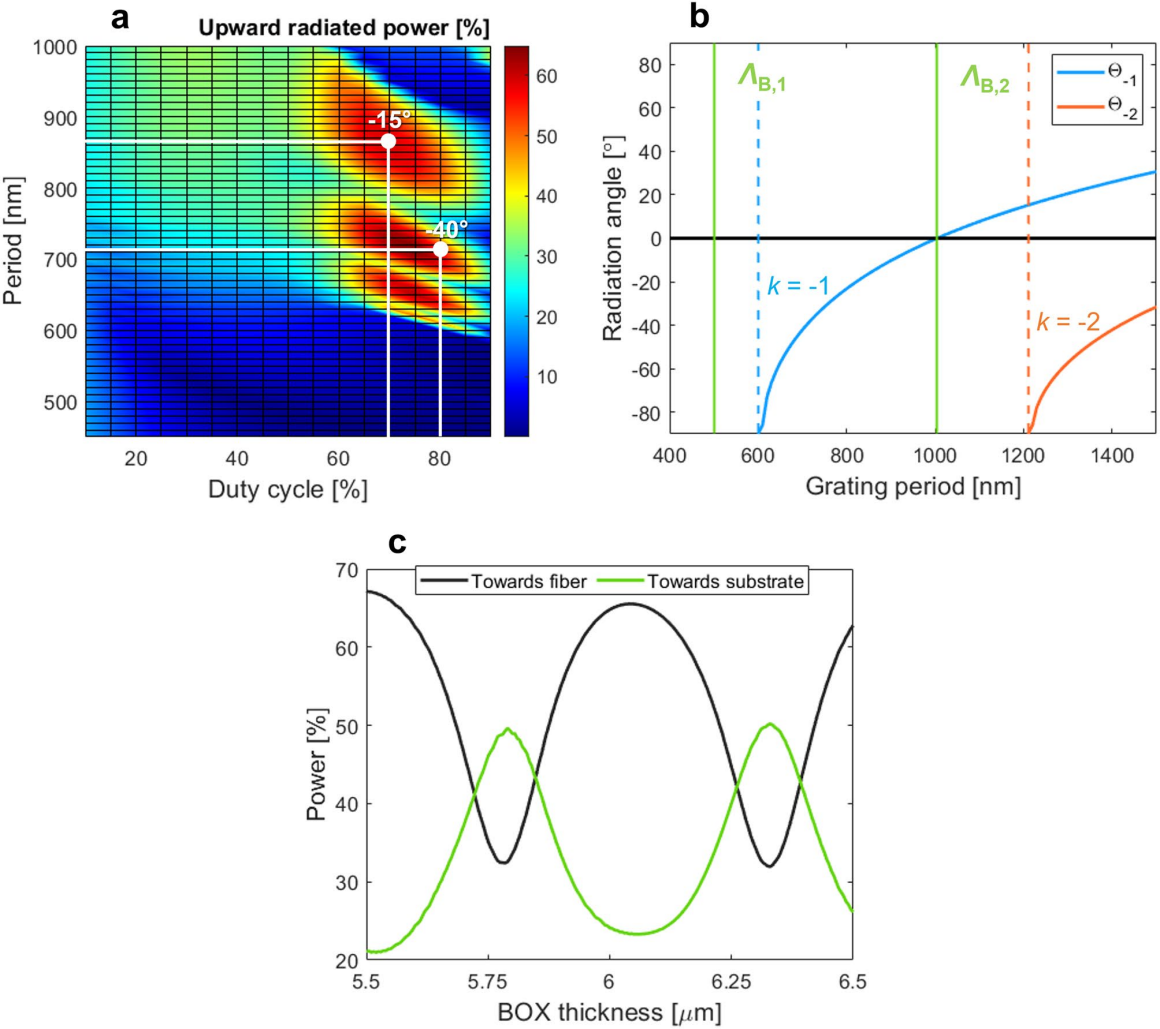
Grating radiation performance at 1.55 μm wavelength. (**a**) Power radiated towards an optical fiber as a function of the duty cycle and grating period. (**b**) Reflection and radiation conditions versus the grating period. For the analysis, the effective index of the fundamental TE-polarized Floquet-Bloch mode was 1.5435, arising from uniform grating design. (**c**) Powers radiated towards fiber and Si substrate as a function of the BOX thickness.

We first designed a uniform grating coupler with fully etched air trenches. Figure 3a shows a 2-D design map of the power radiated towards an optical fiber as a function of the duty cycle and grating period. The period (*Λ*) consists of fully etched trenches and unetched tooth, with lengths *L*_e_ and *L*_n_, respectively. The duty cycle (*DC*) defines the ratio between the length of the unetched tooth and the period.

**Figure 4:**
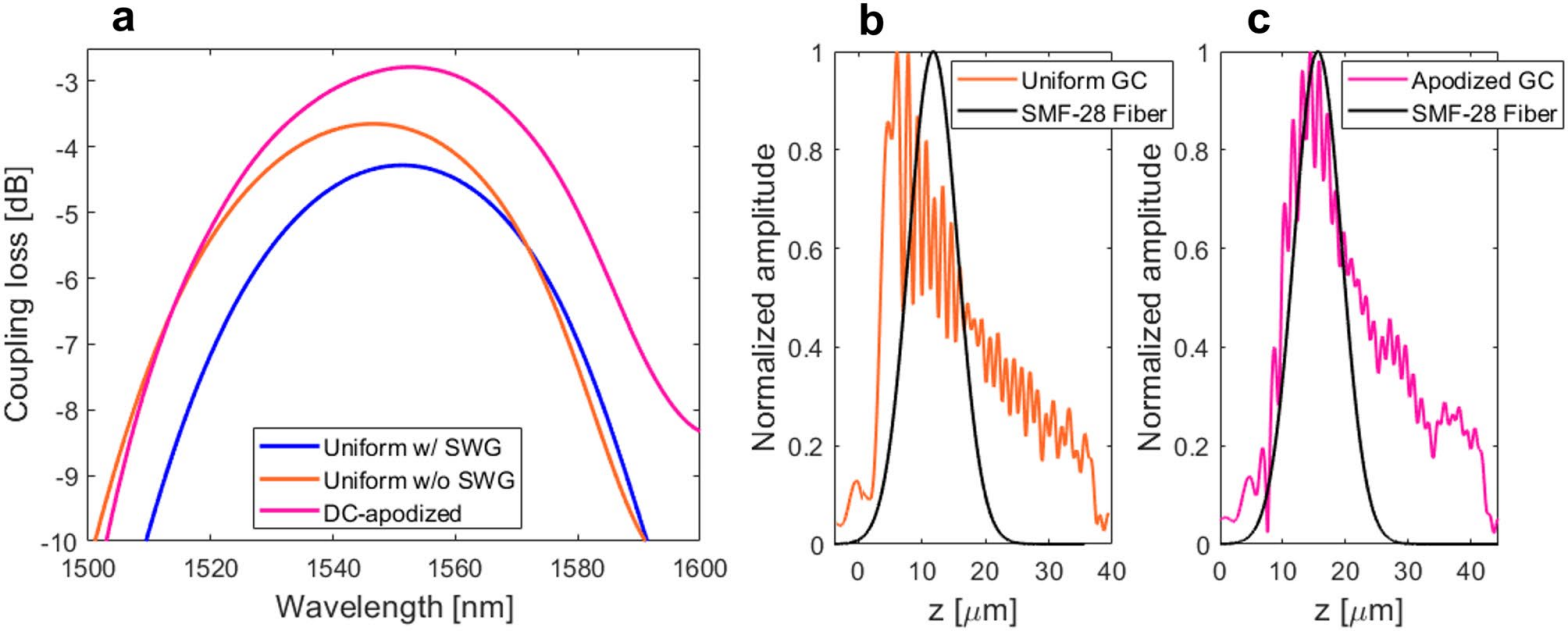
(**a**) Calculated coupling loss as a function of the wavelength for uniform and apodized grating coupler designs. Comparison of the near-Gaussian optical fiber mode and electrical field profiles (dominant *E*_y_ field component) for (**b**) uniform and (**c**) apodized surface grating coupler.

According to our FDTD calculations, the power radiated from the grating to a fiber reaches 63%, while the power lost into the Si substrate is 24%. In turn, the grating directionality, defined as the ratio between the upward radiated power and the total out-radiated power, is 72%. These performances were obtained for a 43 μm long grating, with *Λ* = 860 nm, *DC* = 70%, and − 15° radiation angle tilt from the surface normal direction. A higher directionality (up to 80%) can be found from the 2-D map, however, at the expense of large coupling angle (> − 40°), which is not well-suited for practical testing. The negative coupling angle is selected to favor a single-beam operation (see Fig. 3b), while simultaneously suppressing second-order Bragg reflections and avoiding generation of high-order radiation beams, which otherwise deteriorates the coupling loss. The radiation behavior of the grating is dominated by effects of constructive/destructive interference between the up- and down-radiated beams. Figure 3c shows the periodic evolution of powers radiated towards optical fiber and bottom Si substrate as function of the BOX thickness. The upward power is maximized either by choosing the right BOX thickness or by judiciously adjusting the coupling angle to tune the path length difference between the upward beam and the downward beam reflected at the BOX-to-substrate interface^36–38^. The calculated back-reflections at the waveguide-grating junction is 8% and the near-field overlap between the radiated grating profile and the Gaussian-like fiber mode is 67%. As shown in Fig. 4a, coupling loss of − 3.6 dB, with a 1-dB bandwidth of 39 nm, is predicted for this grating design.

We also designed uniform grating couplers with SWG metamaterials. As schematically shown in Fig. 1b, the air trenches are substituted by SWG nanostructure. The SWG pattern is implemented in the transverse direction (perpendicular to the light propagation) by interleaving non-etched Si_3_N_4_ segments with full-etch air trenches. The structural periodicity (*Λ*_swg_) is shorter than a half of the operating wavelength. All SWG nanostructures are formed in a Si_3_N_4_ layer. The SWG period is chosen as *Λ*_swg_ < *Λ*_Bragg_ = λ/2 *n*_fb_, where *Λ*_Bragg_ is the first-order Bragg period, *λ* is the operating wavelength (*λ* = 1.55 µm, in free space) and *n*_fb_ is the effective index of the fundamental TE-polarized Floquet-Bloch mode in the grating region (here, *n*_fb_ = 1.5435). By controlling SWG geometry, comprising the widths of Si_3_N_4_ blocks (*w*_Si3N4_) and air holes (*w*_Air_), a wide range of equivalent homogeneous metamaterials can be tailored with synthetic refractive index^36–38^. Since the first demonstration of SWG metamaterials in integrated photonics^39–43^, they have been successfully used as a powerful tool for overcoming performance limitations of conventional photonic devices by customizing the light propagation properties^44–46^. SWG-based grating couplers can provide an additional design freedom to optimize the device performance (otherwise limited to *DC* and period variations only), especially by lowering both the back-reflections and grating strength. Whereas reducing the back-reflections is always a desirable feature, further reducing of the grating strength in couplers with moderately small vertical index contrast might seem counterintuitive. Implementing the SWG metamaterials in Si_3_N_4_ grating couplers can provide additional design freedom to control the grating strength, without compromising the single-etch fabrication or requiring complex apodization structures^31^. Designed SWG grating coupler still maintains high-directionality of 76%, with 67% of power radiated into the superstrate and 21% towards the substrate. The surface grating parameters are: *Λ* = 860 nm, *DC* = 70%, *n*_swg_ = 1.30 and radiation angle -13°. The refractive index of the homogenous SWG metamaterial is obtained by treating the SWG nanostructure as a 2-D multilayer slab waveguide in the *x − z* plane and calculating the effective index of its fundamental TE-polarized mode^36^. The SWG layout dimensions are: *w*_air_ = 197 nm, *w*_SiN_ = 203 nm and *Λ*_swg_ = 400 nm. These feature sizes are compatible with standard 193-nm deep-ultraviolet (deep-UV) optical lithography, typically used in silicon photonic foundries. SWG-engineered couplers provide reduced back-reflections of 5% due to the improved index matching at the waveguide-to-grating transition as well as reduced grating strength, which yields decreased grating-fiber field overlap of 56%, as shown in Fig. 4b. Compared to the grating design with air trenches, the field overlap is about 10% lower, resulting in a fiber-chip coupling loss of − 4.3 dB. The spectral performance of SWG-based grating coupler is shown in Fig. 4a, having a 1-dB bandwidth of 38 nm.

The proposed Si_3_N_4_ couplers are further improved by using grating apodization. This is achieved by varying the grating strength along the direction of mode propagation (*z*-direction) using *DC* optimization. Grating apodization enhances the near-field matching between profiles of the radiated grating beam and Gaussian-like fiber mode. The optimization was performed by studying different number of apodized grating periods and various *DC* ranges to gradually vary the grating strength, while keeping critical dimensions larger than 100 nm for compatibility with deep-UV lithography^47^. As a result, the optimal grating coupler has 7 non-uniform period, followed by 43 periods with uniform strength. Beyond this point, the coupling loss starts to saturate and further improvement via grating apodization becomes negligible (see Fig. 10c). In the reference coupler design, the *DC* was linearly varied from 90 to 60% along the 7 periods. To keep the radiation angle constant (− 15°) for all diffraction cells, grating periods were chirped from 810 to 890 nm. According to FDTD calculations, *DC*-apodization improves the field overlap integral to 74% and provides an eight-fold reduction in back-reflections down to 1%. This results in overall coupling loss of − 2.7 dB, with a 1-dB spectral range of 39 nm. The coupling loss as a function of the wavelength is shown in Fig. 4a and corresponding near-field profiles of the optical fiber mode and apodized grating are shown in Fig. 4c.

### Device fabrication and testing

**Figure 5:**
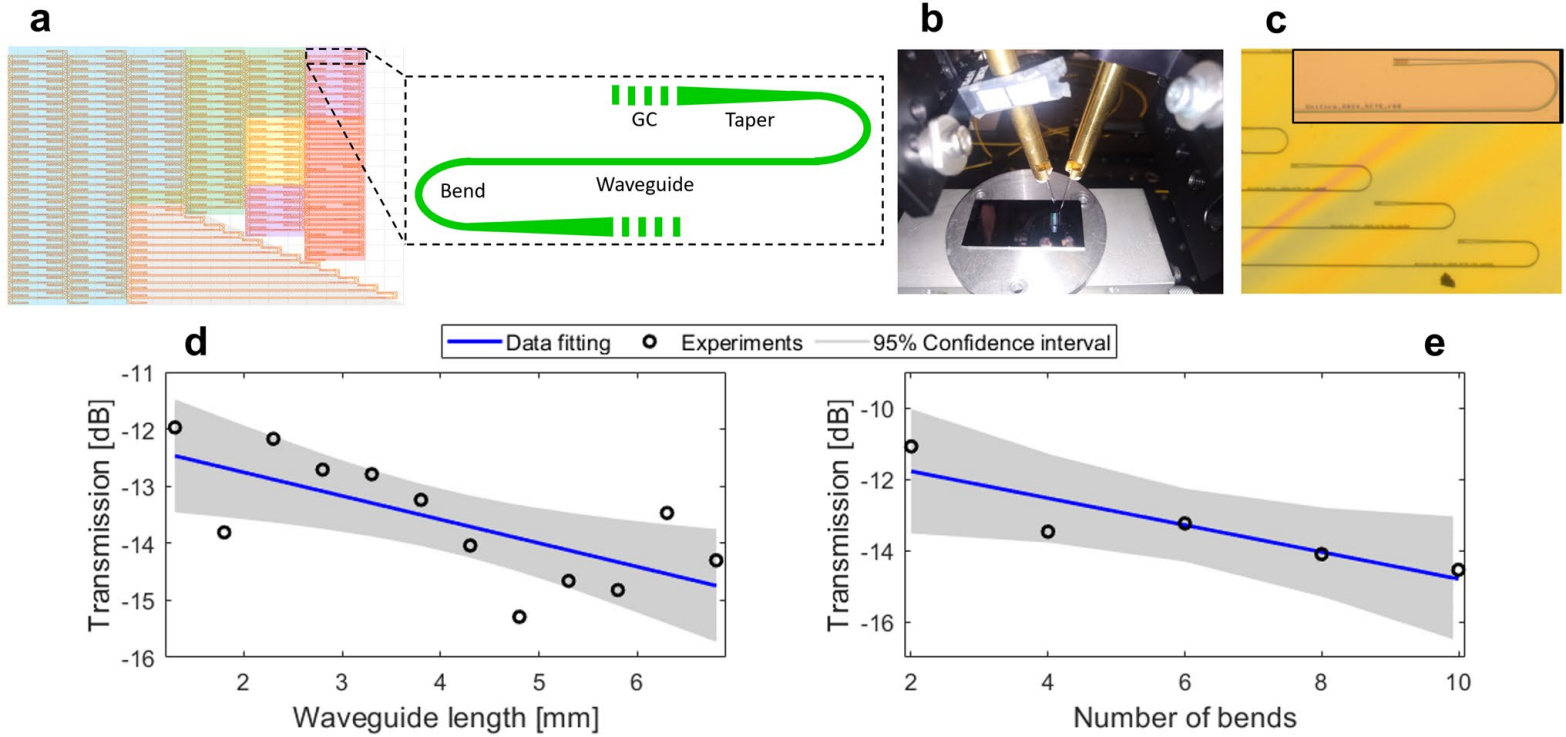
(**a**) Mask layout of Si_3_N_4_ grating couplers. (**b**) Experimental testing set-up. (**c**) Optical microscopy images of fabricated devices. (**d**) Waveguide loss and (**e**) waveguide bend measurements at 1.55 μm wavelength. The solid blue lines are linear fits of the measured points (black dots).

Si_3_N_4_ surface grating couplers were fabricated using single-etch step process on LPCVD wafers, with 400 nm waveguide slab and 6 μm BOX. The resist was patterned by e-beam lithography, followed by reactive ion etching to transfer the device layouts into the Si_3_N_4_ layer. Fabricated devices were characterized using standard back-to-back transmission measurements (detailed in Methods). Waveguide propagation and bending losses of fabricated chips were measured to be 3.0 dB/cm and 0.25 dB/bend, respectively. Measured level of waveguide losses can be attributed to the interface roughness, which consequently increases the scattering losses at the waveguide sidewalls. Reduced waveguide propagation losses can be potentially obtained by covering the samples with oxide claddings to smooth the Si_3_N_4_ film roughness^49,50^ and/or by using customized thermal annealing to get rid of material impurities^9^. Mask layout, testing set-up, and optical microscopy images of fabricated Si_3_N_4_ grating couplers are provided in Fig. 5a–c, while Fig. 5d,e show measurements of propagation and bend losses.

**Figure 6:**
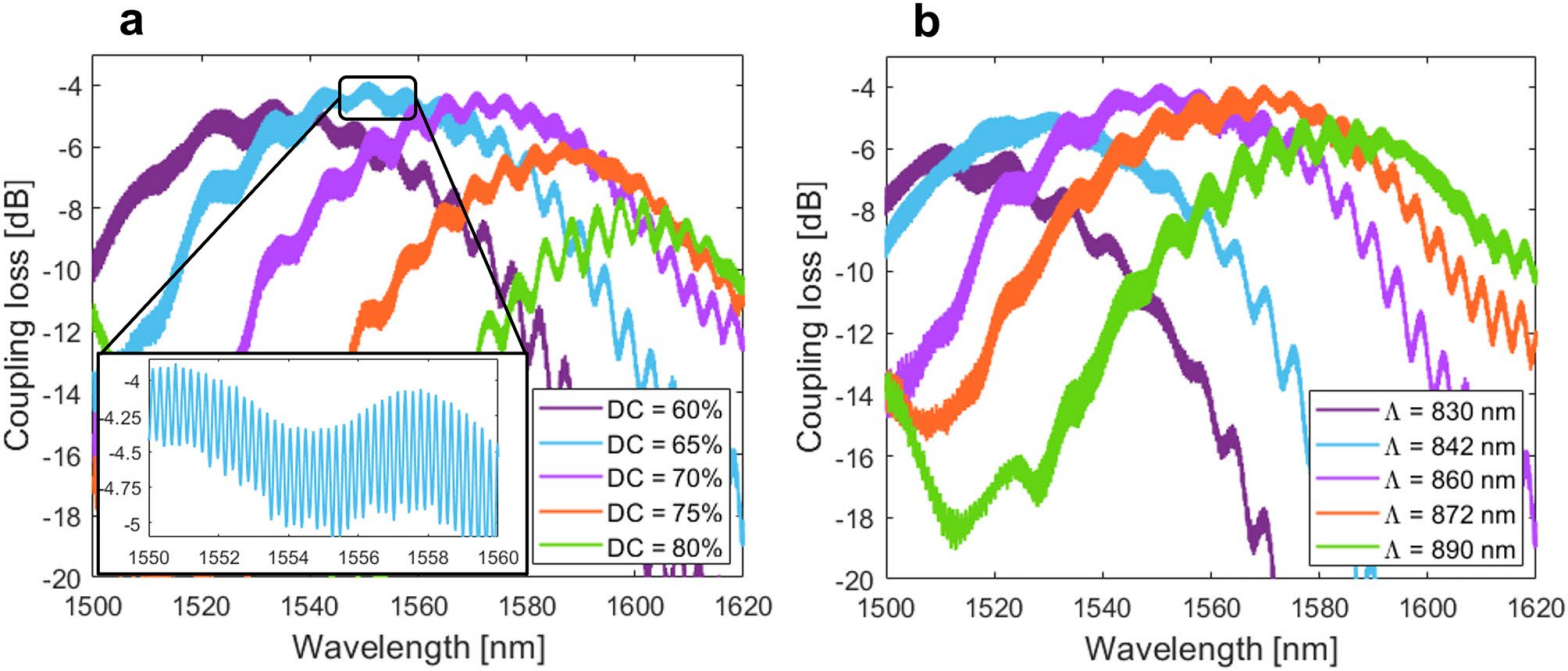
Measured coupling loss as a function of the wavelength for uniform grating couplers with varied (**a**) duty cycles and (**b**) periods. Inset of (**a**): an enlarged view of the high-frequency Fabry–Perot ripples in the measured spectral response.

**Figure 7:**
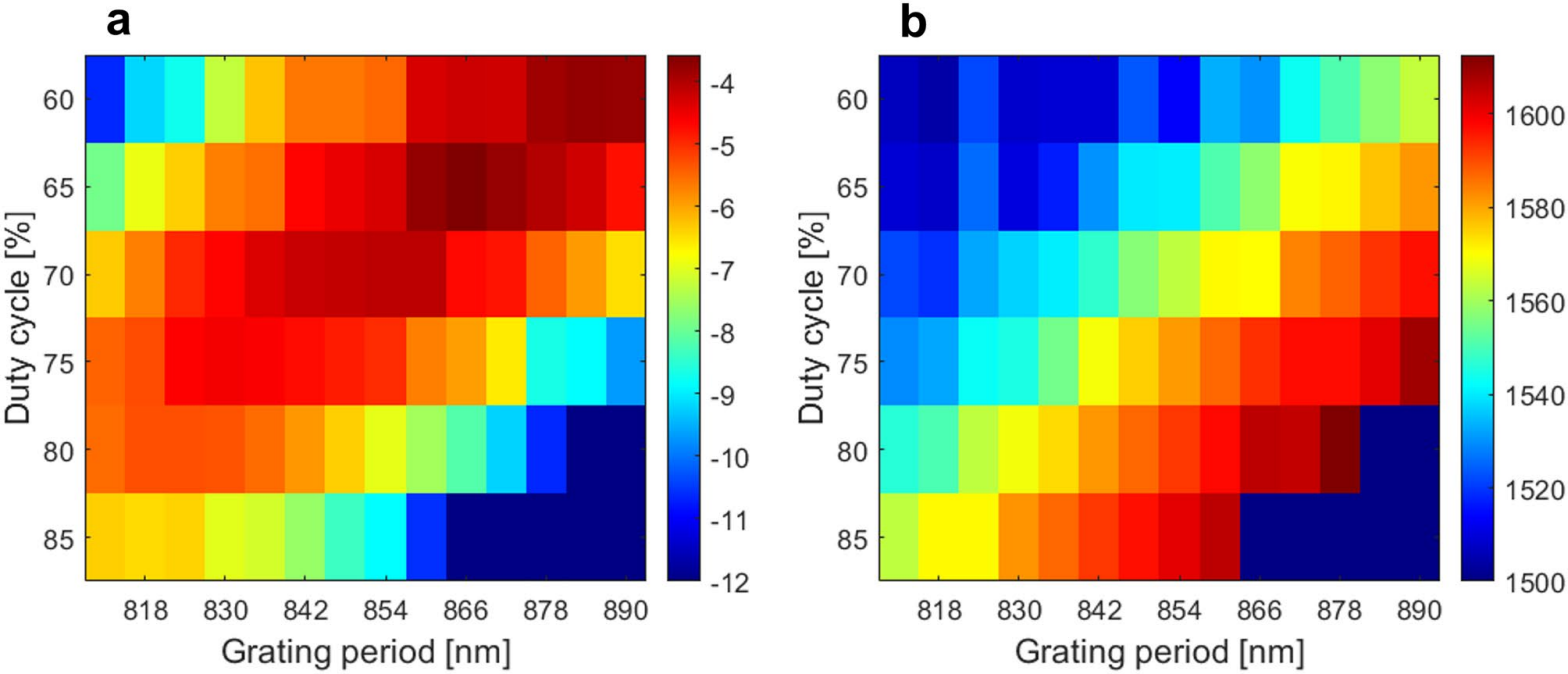
Summary of the measured Si_3_N_4_ unform grating couplers: (**a**) coupling loss and (**b**) central wavelength as a function of the grating period and duty cycle.

**Figure 8:**
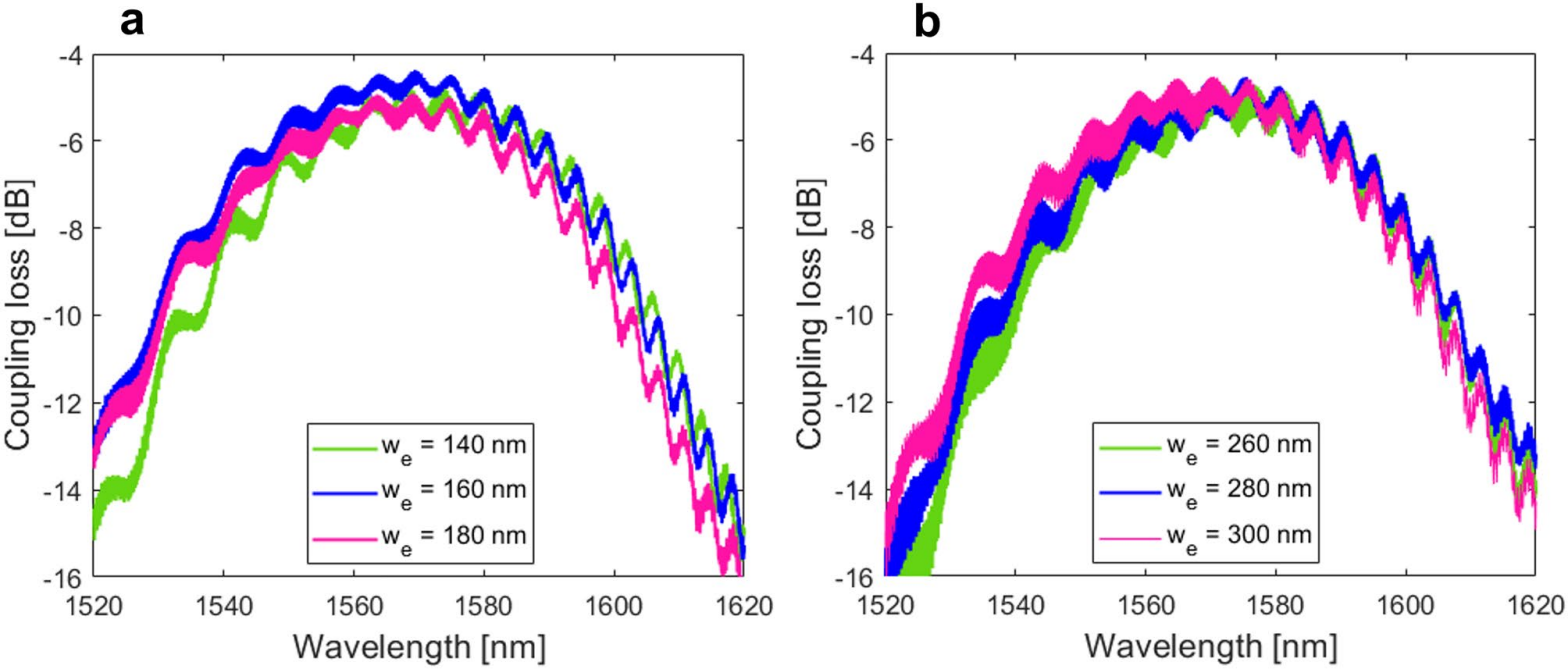
Measured coupling loss as a function of the wavelength for uniform Si_3_N_4_ grating couplers with different transversal SWG geometries. Longitudinal duty cycles are: (**a**) 60% and (**b**) 70%.

**Figure 9:**
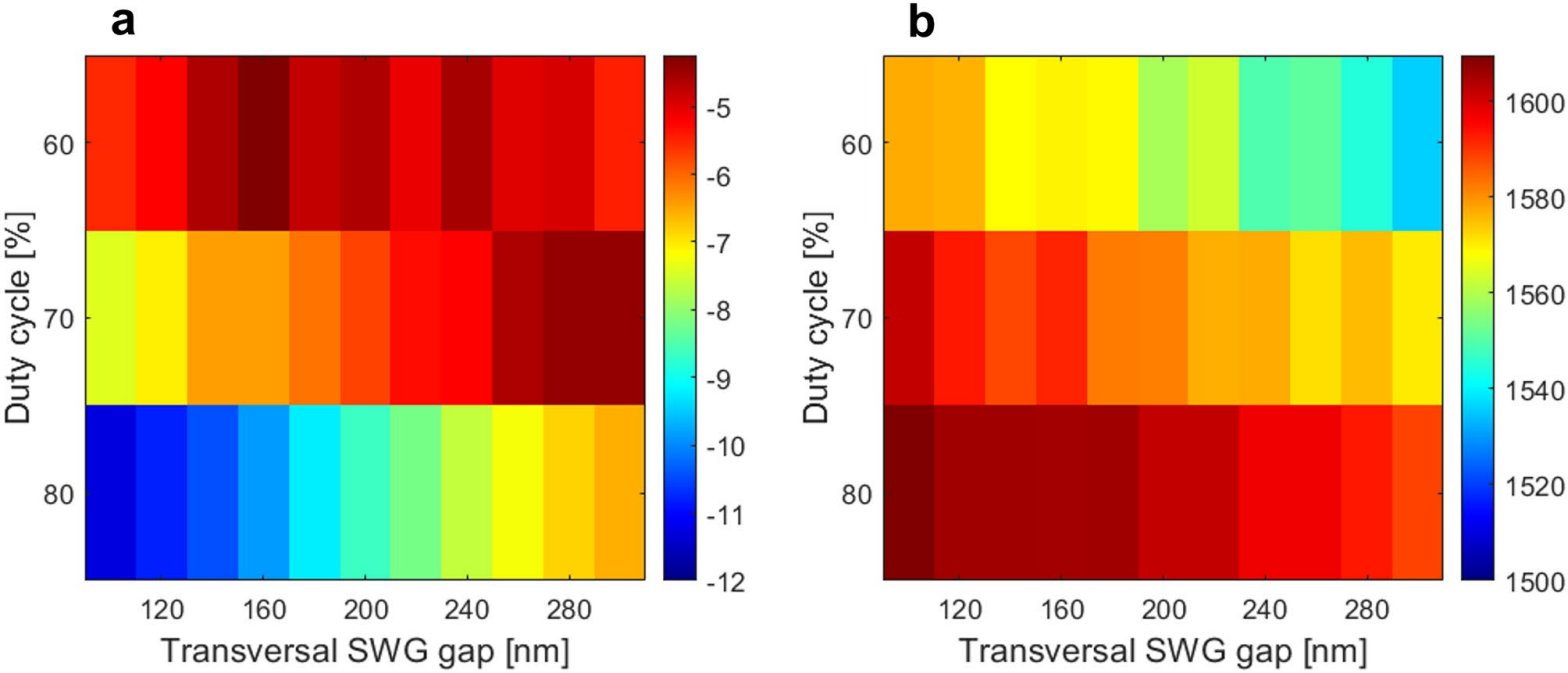
Summary of the measured Si_3_N_4_ uniform grating couplers with SWG metamaterials: (**a**) coupling loss and (**b**) central wavelength as a function of the transversal gap size and duty cycle.

**Figure 10:**
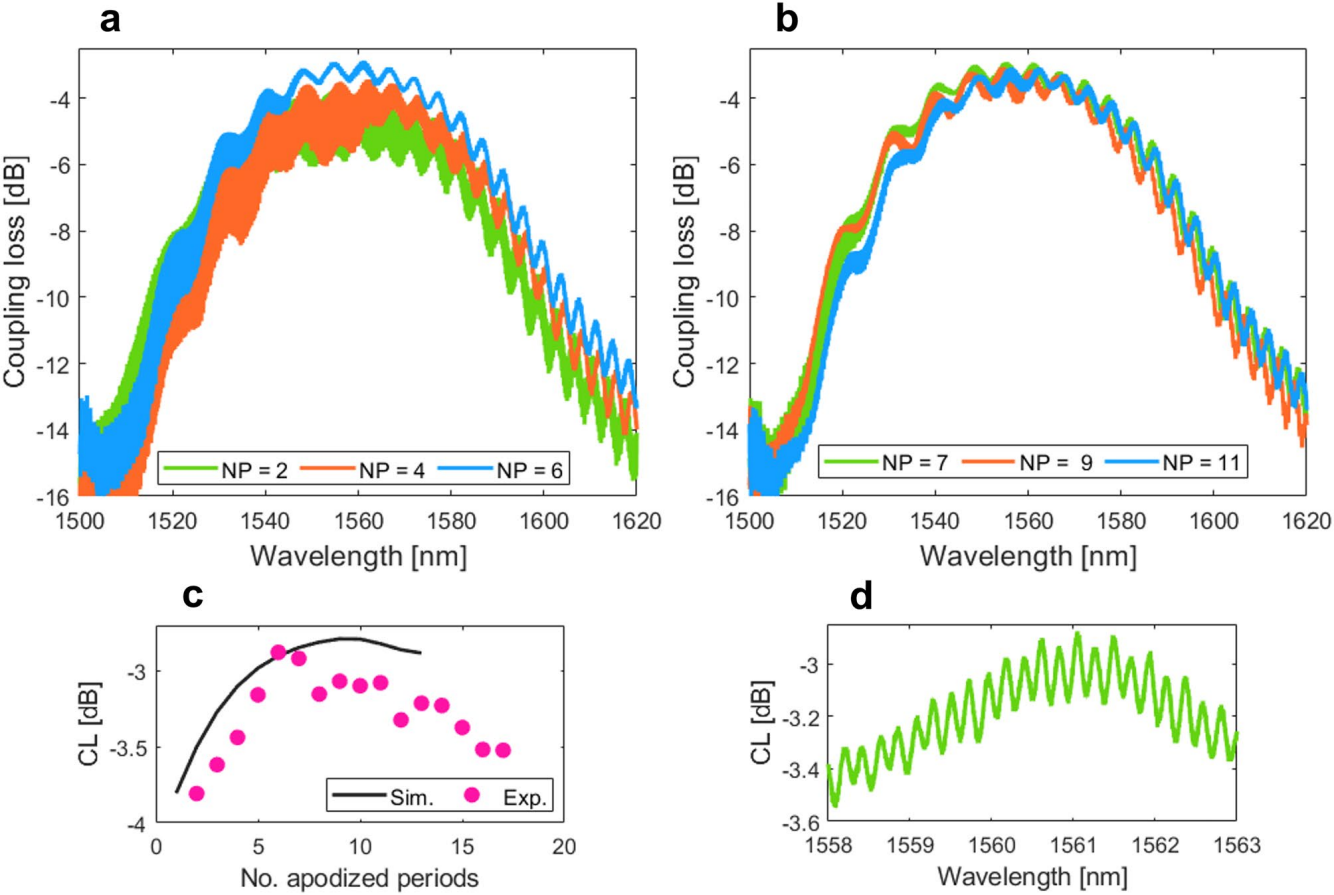
Measured coupling loss as a function of the wavelength for *DC*-apodized grating couplers with varied number of apodized periods: (**a**) NP = 2, 4, and 6, and (**b**) NP = 7, 9, and 11. (**c**) Simulated and experimentally measured coupling loss versus different number of apodized grating periods. (**d**) A magnified view of the Fabry–Perot fringes for the grating coupler with 7 apodized periods.

The experimental results obtained in Si_3_N_4_ surface grating couplers are comprehensively presented in Figs. 6, 7, 8, 9,10. In particular, Fig. 6a,b show the measured coupling loss as a function of the wavelength for uniform gratings with varied duty cycles and periods. Devices were characterized with a radiation angle of -15°. The peak coupling loss of − 3.9 dB centered at 1550 nm wavelength was measured for a grating coupler with 860 nm long period and duty cycle of 65%. The experimental loss is only 0.3 dB higher than the design prediction of − 3.6 dB. This is in excellent agreement with FDTD simulations, taking into the account − 5% bias in the duty cycle. As observed in Fig. 6a, intended variation in duty cycle shifts the grating spectral response towards longer (positive bias) and shorter (negative bias) wavelengths. Observed trends agree well with the grating theory governed by the phase matching condition given by Eq. (1). Higher (lower) duty cycle increases (decreases) the effective index of the fundamental TE-polarized Bloch-Floquet mode inside the grating, and for a fixed fiber angle and period, the peak wavelength is either red- or blue-shifted. Specifically, for a duty cycle variation of ± 5%, corresponding central wavelengths are shifted of about ± 21 nm to 1571 nm and 1534 nm, respectively. From a geometrical perspective, this duty cycle offset translates into the grating trench/tooth length changes of about ± 43 nm. The peak coupling loss associated with these variations are found to be − 4.2 dB and − 4.5 dB, yielding a loss penalty in a 0.3–0.6 dB range only. This clearly indicates that the designed grating couplers are robust to fabrication imperfections, mainly due to the errors in device pattern definition and etching. Moreover, for variations in grating periods, shown in Fig. 6b, we observed noticeable spectral shifts in the measured spectral response. This observation is also accompanied by higher coupling losses. In particular, for a period variation of ± 30 nm, the peak wavelengths are displaced to 1511 nm (for 830 nm long period) and 1582 nm (for 830 nm long period), with fiber-chip coupling losses of − 5.9 dB and − 4.9 dB, respectively. Corresponding coupling loss penalties are substantially higher, ranging from 1 to 2 dB compared to the nominal device. In this case, the higher coupling losses for deviated devices are caused by reduced directionality (less power is radiated towards a fiber) and higher field overlap mismatch between the grating beam and fiber mode. As the grating directionality is maximized through the angle adjustment, instead of BOX thickness optimization, negative and positive offsets from the reference period reduce the amount of power radiated from the grating (see map of Fig. 3a). In addition, variation in the grating period also strongly impacts the field overlap. The grating-fiber overlap is typically maximized locally for single (central) wavelength and optimal (in practice fixed) radiation angle. However, the field overlap deviates quickly from the nominal value due to the resonant nature of the surface grating, which introduces strong wavelength-dependence of the radiation angle. Figure 7a,b sum up experimental measurements of more than 80 uniform surface grating couplers.

It can be observed that the measured spectral response of the uniform grating couplers has both high- and low-frequency ripples. These ripples can be linked with two main sources. The low-frequency spectral fringes are due to the Fresnel reflections occurring at the surface of the fiber. This reflection loss originates from the fact that surface grating couplers were tested with air only. To preclude this loss source, the index matching liquids can be used to lower back-reflections at the grating-to-air and air-to-fiber interfaces^24,32^. On the other hand, the high-frequency ripples in the grating spectrum, shown in the inset of Fig. 6a, are caused by the back-reflections due to the residual index mismatch at the junction between the diffraction grating and Si_3_N_4_ slab waveguide. The magnitude of the ripples shown in the inset varies from 0.7 to 0.9 dB, resulting in grating back-reflection of ~ 7%. From other spectral responses, it is observed that larger duty cycles reduce the magnitude of the high-frequency Fabry–Perot fringes as a result of reduced discontinuity (and thus improved index matching) between the fundamental TE modes of the Si_3_N_4_ injection waveguide and the surface grating. To effectively mitigate this back-reflections, established approaches such as grating apodization^23,24^ or curving and offsetting the grating lines^19,31,51^ can be utilized.

Figure 8 shows the coupling loss as a function of the wavelength for uniform surface gratings with SWG metamaterials and different longitudinal duty cycles of 60% and 70%, respectively. The back-to-back transmission between input and output gratings was maximized, considering − 13° fiber angle. As shown in Fig. 8a,b, the peak coupling loss was − 4.4 dB and − 4.6 dB at a wavelength of 1570 nm for respective duty cycles. The measured spectral response was shifted towards longer wavelengths of about 20 nm. Compared to the reference design of uniform grating with SWG metamaterials (− 4.3 dB loss), associated fiber-chip loss penalties were 0.1–0.3 dB only. Furthermore, considering intended air gap variations of ± 20 nm (for both duty cycle designs), measured fiber-chip coupling loss shows relatively small deviations, ranging from 0.2 to 0.6 dB.

Moreover, experimental measurements also confirmed that the SWG-based Si_3_N_4_ couplers have higher coupling loss than their air-based counterparts. Indeed, this is a result of reduced grating strength, and thereby reduced grating-fiber field overlap. Si_3_N_4_ grating couplers with SWG metamaterials, that are here demonstrated for the first time, also showed an improved tolerance to fabrication errors, while providing a further potential to leverage SWG engineering in the development of efficient grating couplers. In total, over 30 Si_3_N_4_ grating couplers with SWG nanostructure were measured. The coupling losses and central wavelengths as a function of the duty cycle and the transversal SWG gap width are summed up in Fig. 9a,b, respectively.

Figure 10a,b show the coupling loss as a function of the wavelength for *DC*-apodized surface grating couplers in SiN waveguide platform with a varied number of apodized periods. We measured a minimum fiber-chip coupling loss of − 2.9 dB at a wavelength of 1561 nm for a grating design with 7 apodized periods. This is a 1 dB coupling loss improvement compared to the uniform grating coupler shown in Fig. 6. Moreover, comparing experimental results with FDTD design calculations, we achieved an excellent agreement, yielding only a marginal—0.2 dB high—loss difference with respect to the nominal design of − 2.7 dB. Figure 10c compares simulation results (solid black line) and retrieved fiber-chip coupling loss (magenta hexagons) both as a function of the number of apodized grating periods. In total, 15 device flavors were tested. We can clearly observe identical trends between the theory and experiments. The overall loss difference between designs and measurements in tested devices is related to the presence of fabrication errors. More specifically, variations in lengths of the etched trenches and unetched tooth coupled with small differences in the grating period, deteriorate the amplitude profile of the out-radiated grating beam. This aspect is particularly critical within the apodized grating section. As a consequence, the mismatch between the near-field grating beam and the Gaussian-like optical fiber mode profile is higher, hence the coupling loss as well. This observation is also supported by the data provided in Fig. 10c. The loss penalty becomes higher for grating couplers with a larger number of apodized periods. This effect becomes especially pronounced for devices, having more than 10 apodized periods. Last, but not least, Fig. 10d shows an enlarged view on the spectral response of the grating coupler with 7 apodized periods. It can be observed that the magnitude of high-frequency Fabry–Perot ripples is substantially lower than it was for uniform grating coupler (see inset of Fig. 6a). In a good agreement with design, the grating apodization creates a smooth transition between the input waveguide and the surface grating by gradually reducing the residual index mismatch between individual grating cells. From the magnitude of the Fabry–Perot ripples, we estimated the back-reflections of ~ 3%.

## Conclusion

We have demonstrated a library of fabrication-robust Si_3_N_4_ grating couplers with low coupling losses and facile single-etch step fabrication. Experimentally, at *C*-band wavelengths, the fiber-chip coupling losses of − 4.4 dB and − 3.9 dB were obtained for uniform designs and − 2.9 dB loss were measured for apodized grating couplers, in both cases obviating the complex fabrication based on backside reflectors, top grating over-layers, or multi-level configurations. In total, more than 130 grating couplers were comprehensively characterized, showing an excellent agreement between designs and experiments and demonstrating error-tolerant performance to the presence of fabrication imperfections. Our results pave a promising way towards the development of low-loss, error-tolerant, and cost-effective grating-coupled optical interfaces. Demonstrated off-chip grating couplers may prove essential for establishing versatile and scalable Si_3_N_4_ photonic integrated circuits for optical communications or quantum information sciences.

## Methods

### Device designs

Design and analysis of surface grating couplers and interconnecting waveguides was carried out by using full-vectorial *Ansys Lumerical* toolset, including two-dimensional (2-D) finite difference element (FDE) and three-dimensional (3-D) eigenmode expansion (EME) and finite difference time domain (FDTD) solvers, respectively. The 2-D FDE and 3-D EME calculations were performed to design chip interconnecting structures (single-mode waveguides, waveguide bends, tapers), while 2-D and 3-D FDTD simulations were employed for designing variety of surface grating couplers. The window sizes for 2-D and 3-D FDTD simulations were 5.4 μm × 50 μm (*y* and *z* directions) and 5.4 μm × 13.5 μm × 50 μm (*y*, *x*, and *z* directions), respectively. The uniform mesh size used for all simulations was *Δx* = *Δy* = *Δz* = 10 nm, with a corresponding simulation time step satisfying the Courant-Friedrichs-Lewy convergence condition.

### Fabrication

Surface grating couplers and interconnecting waveguides were fabricated using low-pressure chemical vapour deposition (LPCVD) silicon nitride (Si_3_N_4_) photonic platform, with a waveguide core thickness of 400 nm and 6 μm thick buried oxide (BOX) layer. Electron beam (e-beam) lithography was used to define waveguide and surface grating patterns using the single mask layer. Patterns were transferred into the Si_3_N_4_ layer by dry reactive ion etching, with BOX acting as etch stop layer.

### Experimental testing

Optical characterization was performed by a pair of identical surface grating couplers connected in a back-to-back configuration. Input and output couplers with 15 μm transverse width were connected through 400 μm long tapers, waveguide bends of 80 μm radius, and 1-μm-wide single-mode waveguides. The light generated by a tunable *C*-band optical source was coupled by cleaved fibers into the Si_3_N_4_ chip via one grating and coupled out by the second grating. Transmission between the surface gratings was maximized for a transverse electric (TE) polarization using a polarization controller. The fiber-chip coupling loss was determined from the measured transmission of two back-to-back connected grating couplers, subtracting the losses of waveguides, bends, and test set-up. Waveguide propagation loss and bend loss were determined by using test structures with varying waveguide length (Fig. 5d) and number of bends (Fig. 5e), respectively. Both losses were extracted by using the slope of the measured transmission function, including R-squared model and 95% confidence interval. The set-up loss was determined from a fiber-to-fiber calibration tests, excluding the Si_3_N_4_ chip between the fibers, while optical fibers, polarization controller and fiber connectors were included. The set-up loss was 1.5 dB. The fabricated chips were measured with air as a superstrate medium and with SMF-28 optical fibers without AR coating. The samples were tested without the use of index matching liquid.

## Data Availability

The data underlying the results presented in this paper are available from the Authors upon reasonable request.
